# Biomonitoring of marine vertebrates in Monterey Bay using eDNA metabarcoding

**DOI:** 10.1371/journal.pone.0176343

**Published:** 2017-04-25

**Authors:** Elizabeth A. Andruszkiewicz, Hilary A. Starks, Francisco P. Chavez, Lauren M. Sassoubre, Barbara A. Block, Alexandria B. Boehm

**Affiliations:** 1 Department of Civil and Environmental Engineering, Stanford University, Stanford, CA, United States of America; 2 Center for Ocean Solutions, Stanford University, Stanford, CA, United States of America; 3 Monterey Bay Aquarium Research Institute, Moss Landing, CA, United States of America; 4 Department of Biology, Hopkins Marine Station, Stanford University, Pacific Grove, CA, United States of America; University of Hyogo, JAPAN

## Abstract

Molecular analysis of environmental DNA (eDNA) can be used to assess vertebrate biodiversity in aquatic systems, but limited work has applied eDNA technologies to marine waters. Further, there is limited understanding of the spatial distribution of vertebrate eDNA in marine waters. Here, we use an eDNA metabarcoding approach to target and amplify a hypervariable region of the mitochondrial 12S rRNA gene to characterize vertebrate communities at 10 oceanographic stations spanning 45 km within the Monterey Bay National Marine Sanctuary (MBNMS). In this study, we collected three biological replicates of small volume water samples (1 L) at 2 depths at each of the 10 stations. We amplified fish mitochondrial DNA using a universal primer set. We obtained 5,644,299 high quality Illumina sequence reads from the environmental samples. The sequence reads were annotated to the lowest taxonomic assignment using a bioinformatics pipeline. The eDNA survey identified, to the lowest taxonomic rank, 7 families, 3 subfamilies, 10 genera, and 72 species of vertebrates at the study sites. These 92 distinct taxa come from 33 unique marine vertebrate families. We observed significantly different vertebrate community composition between sampling depths (0 m and 20/40 m deep) across all stations and significantly different communities at stations located on the continental shelf (<200 m bottom depth) versus in the deeper waters of the canyons of Monterey Bay (>200 m bottom depth). All but 1 family identified using eDNA metabarcoding is known to occur in MBNMS. The study informs the implementation of eDNA metabarcoding for vertebrate biomonitoring.

## Introduction

Stressors such as ocean acidification, overfishing, coastal development, pollution, and changes in sea surface temperature can cause loss of biodiversity and shifts in species distributions within marine and estuarine environments [1–5]. The rate of species extinction is higher today than it was in pre-human periods and the introduction of invasive species has changed the structure and function of ecosystems [4,6,7]. The National Oceanic and Atmospheric Administration (NOAA) currently lists 2,270 marine species as endangered or threatened under the Endangered Species Act and estimates an annual cost of $137 billion for control and eradication of marine or estuarine invasive species [8,9]. Oceanographic efforts to measure organism abundance and distributions are often conducted annually using shiptime surveys, electric and conventional tags, nets, and ROVs and in some cases can be harmful to species or habitats. New data collection methods are needed to better understand changes in organismal abundance, biodiversity and community structure over shorter time scales [6,10].

Recent advances in metagenomics suggest that the presence and abundance of aquatic vertebrates and invertebrates can be determined by analyzing environmental DNA (eDNA) extracted from water samples [11]. Because of its non-invasive nature, and the relative ease of water sampling, using eDNA for biomonitoring in aquatic systems could enable the acquisition of temporally and spatially intensive biodiversity data sets [6,12]. eDNA is DNA that has been shed from organisms and then retained in environmental matrices such as soil or water. eDNA from macroorganisms may originate from feces, mucus, blood, and sloughed cells, tissue, or scales; in some cases, it may be attached to particles [10,13,14]. Early studies investigated ancient eDNA preserved in soil matrices [15–19]. More recently, methods have been applied to capture contemporary eDNA in sediment [20] and in aquatic matrices by filtering or precipitating eDNA from water [21]. eDNA captured on a filter or precipitated from water samples can be PCR-amplified using either species-specific or “universal” primers. Amplification using species-specific primers in conjunction with a hydrolysis probe yields quantitative results regarding gene copy concentration via real-time quantitative PCR (qPCR). To conduct a community analysis, eDNA can be PCR-amplified with “universal” primers (e.g. targeting the mitochondrial 12S rRNA gene, 16S rRNA gene, 18S rRNA gene) and then sequenced using next generation sequencing (NGS) [22–24]. Universal primers are developed by aligning whole genomes of groups of species of interest (e.g., bony fishes, marine vertebrates) and optimizing primers to target a short region of a gene that is evolutionarily conserved among all species in the group, but varies enough within that region among species to correctly identify taxa to the genus or species level [23]. Each environmental sample gets a unique “tag” (6 base pairs) added to the primers during PCR amplification, making it possible to sequence several samples on one sequencing run and to separate samples post-sequencing. This significantly reduces the cost of sequencing and is commonly referred to as “eDNA metabarcoding”.

The power of NGS for marine biomonitoring is already being realized. For example, the TARA expeditions conducted large-scale global sampling and the associated researchers have analyzed more than 7 terabases of metagenomic data. They have focused primarily on viruses, prokaryotes, and picoeukaryotes, and the researchers have not yet investigated the use of eDNA to identify macroorganisms [25,26]. Other researchers have applied eDNA methods for identifying macroorganisms and have shown that under some conditions, eDNA methods can more accurately identify species by avoiding biases inherent to traditional biomonitoring methods (e.g., misidentification or lack of identification in visual surveys) [10,27–30]. Though results from previous studies provide evidence that eDNA methods hold promise as sound biomonitoring tools, questions still remain about how to properly sample water and interpret sequencing results [31,32] and few studies [2,10,23,33–36] have investigated the feasibility of using eDNA methods to detect macroorganisms in marine waters.

The present study uses eDNA metabarcoding to census marine vertebrates in the Monterey Bay National Marine Sanctuary (MBNMS). This study expands on a previous eDNA metabarcoding study conducted along a short, 2.5 km transect within MBNMS [33] by extending the spatial scale to 45 km. We use a recently published universal primer set (MiFish-U) [23] that targets fish for eDNA metabarcoding. The objectives of our work are to: (i) investigate whether eDNA metabarcoding identifies spatial differences between vertebrate communities present in MBNMS, (ii) compare operational taxonomic units (OTUs) identified using eDNA metabarcoding across biological replicates, and (iii) compare the eDNA metabarcoding census with historical records of species known to occur in MBNMS. To date, there have been seven studies published that use eDNA metabarcoding to identify vertebrates in marine water and some of these studies used microcosms and not actual environmental waters [2,10,23,33–36]. Given the paucity of studies applying eDNA metabarcoding to environmental oceanic samples, this study provides additional proof of concept needed to inform the potential implementation of eDNA metabarcoding for biomonitoring.

## Materials and methods

### Sample collection and laboratory processing

**Fig 1:**
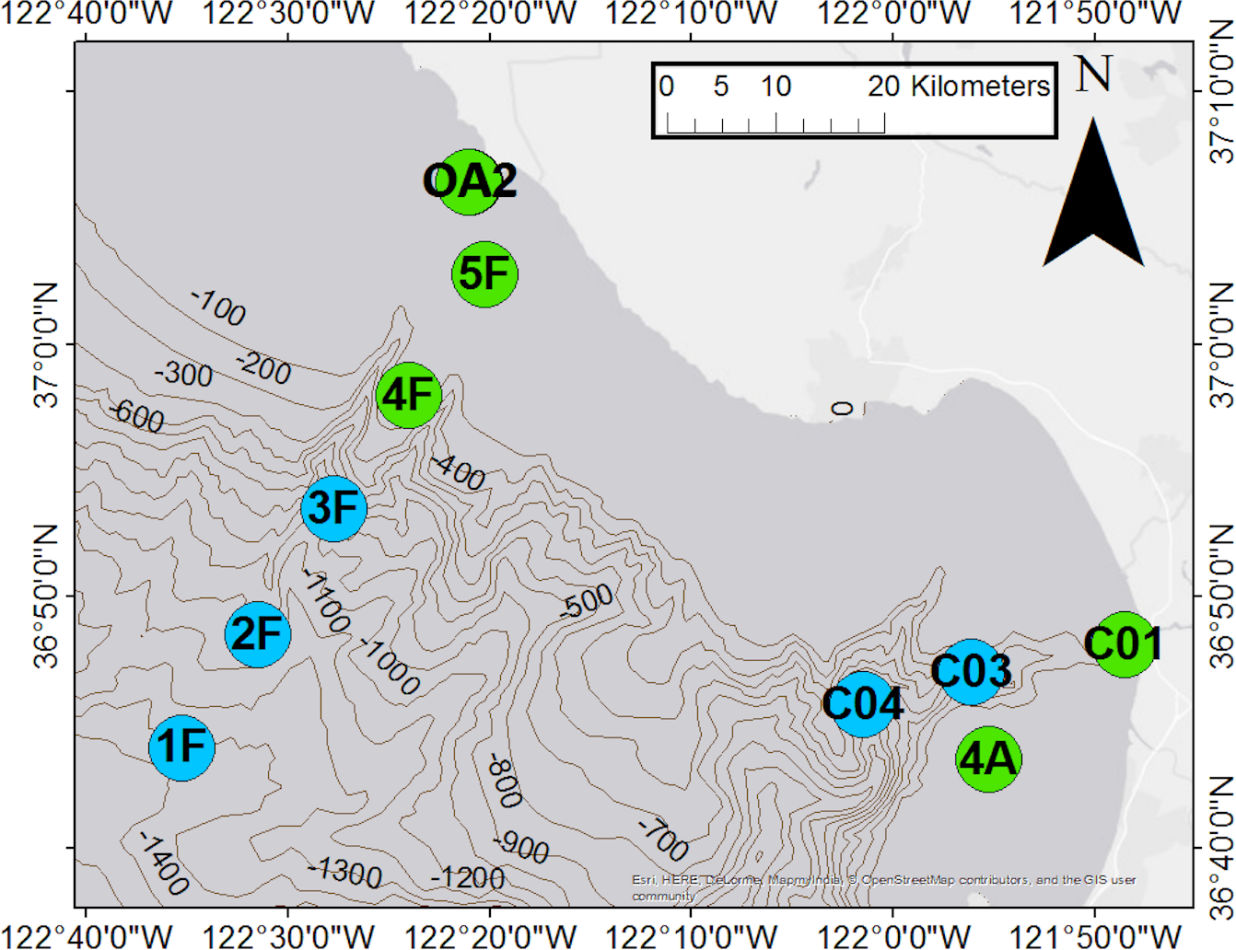
Stations Sampled for eDNA metabarcoding analysis. All are located within the Monterey Bay National Marine Sanctuary. See Table 1 for bottom depth of each station; green stations are on the shelf (<200 m water column depth) and blue stations are in a canyon (>200 m water column depth). Isobaths are labeled with water column depth in meters.

We collected seawater in MBNMS from 29 September 2015 to 1 October 2015 from the Monterey Bay Aquarium Research Institute (MBARI) R/V *Western Flyer*, as a part of MBARI’s Controlled, Agile, and Novel Ocean Network (CANON) project. Research activities in the MBNMS are covered in an annual permit to MBARI by MBNMS. The CANON cruise activities are included in the 2015 permit. The water samples collected and processed as part of this study are not subject to any other permit requirement and no other disposition is required. No approval was needed from the Institutional Animal Care and Use Committee because our methods collect water samples with eDNA shed from vertebrates, not vertebrates themselves. We sampled ten stations at two depths, with one station (OA2) being sampled on two days (Fig 1, Table 1).

**Table 1:** Sample collection metadata.

Date	Time	Station Name	Lat. (N)	Long. (W)	Water Column Depth (m)	Sampling Depth (m)	ΔT Between Surface and Subsurface (°C)	Biological Replicates
9/29/15	12:00	C01	36 48.100	121 48.510	137.3	0	-2.35	3
						40		3
9/29/15	14:32	C03	36 46.990	121 56.110	691.7	0	-2.45	3
						40		3
9/29/15	15:30	C04	36 45.709	122 01.510	919.7	0	-3.31	3
						40		3
9/29/15	20:46	1F	36 43.999	122 35.269	2432.3	0	-3.45	3
						40		3
9/29/15	23:16	2F	36 48.469	122 31.511	1885.1	0	-3.34	3
						40		3
9/30/15	0:37	3F	36 53.479	122 27.761	1500.2	0	-2.82	3
						40		3
9/30/15	3:10	4F	36 57.990	122 24.011	195.8	0	-3.55	3
						40		3
9/30/15	4:51	5F	37 2.760	122 20.261	71.6	0	-3.03	3
						40		3
9/30/15	9:15	OA2	37 6.449	122 21.000	26.8	0	-1.38	3
						20		3
9/30/15	18:36	4A	36 43.519	121 55.250	95.7	0	-2.43	3
						40		3
10/1/15	15:20	OA2	37 6.419	122 21.030	27.5	0	-1.38	3
						20		3

No shading indicates stations on the shelf (<200 m water column depth). Shading indicates stations within a canyon (>200 m water column depth).

At each station, two depths were sampled; the surface (0 m) and subsurface (20 or 40 m). At each depth, three 1 L samples were collected using a 12-bottle rosette sampler. The three samples were collected in distinct niskin bottles and represent true biological triplicates. Each 1 L sample was transferred from the niskin into a new, polycarbonate single-use sterile, disposable bottle. Samples were vacuum-filtered onto 0.22 μm pore size (47 mm diameter) Durapore polyvinylidene flouride filters (Millipore, USA) using 250 mL disposable analytical test filter funnels filled four times (Nalgene, USA). This resulted in 66 environmental samples (three replicates per sampling depth, two sampling depths per station, ten stations with one station sampled twice). Filtration blanks (n = 3) were created by filtering 1 L of deionized water in the same manner as the environmental samples to check for contamination during field collection. Filters were immediately placed in sterile 5 ml plastic scintillation tubes and stored at -80°C for the remainder of the cruise (until 5 October 2015). The samples were transported to the lab post-cruise on dry ice and stored at -80°C until extraction within 2 months of collection.

For this study, we refer to the location where sampling was conducted as a “station” (i.e., 1F, 2F, etc.), a depth at a station as a “sampling depth” (i.e., 0 m or 20/40 m, surface or subsurface), and each biological replicate (1 L of water filtered) a “sample”. We therefore collected 3 samples at each sampling depth, and 2 sampling depths at each of the 10 stations.

#### Laboratory environment

Processing was performed at Stanford University. Benchtops were cleaned with 10% bleach for 10 minutes and then wiped with 70% ethanol. Benchtops were wiped with RNASE AWAY before beginning molecular work. Pipettes were wiped with RNASE AWAY and UV-irradiated for at least 10 minutes before use. DNA extractions were performed on one bench, PCR preparation was performed in a designated DNA-free hood, PCR amplification was performed in a separate room in the laboratory, and post-PCR work was performed in yet another separate room.

#### DNA extraction

We performed extractions in 6 sets, adding in an extraction blank (extraction reagents added to an empty 5 mL tube with no filter, n = 6) for each extraction set. Samples were randomized prior to extraction. We extracted DNA from each filter using the DNeasy Blood and Tissue Kit (Qiagen, USA) following the manufacturer’s protocol with the following modifications. We added 850 μL of lysis buffer [37], 100 μL of SDS (final concentration (C_f_) = 1%), and 100 μL of proteinase K (Qiagen, USA) (C_f_ = 1 mg/mL) to each filter and incubated at 56°C for 14–16 hours. After incubation, we added 1 mL of Buffer AL (Qiagen, USA) and incubated at 56°C for 10 minutes. Then we added 1 mL of 100% molecular grade ethanol and mixed thoroughly by vortexing. We loaded the lysate from each filter into spin columns and used a QIAvac 24 Plus (Qiagen, USA) vacuum manifold. Luer plugs were soaked in 10% bleach and rinsed with deionized water before each use. After loading the 3 mL of lysate, we followed the DNeasy Blood and Tissue Kit protocol. We performed 2 elutions of 50 μL each for a total extract volume of 100 μL. We immediately quantified total DNA using the QUBIT DSDNA HS ASSAY (Invitrogen, USA) and stored extracts at -20°C until amplification within 1 month of extraction.

#### PCR amplification

In addition to the environmental samples (n = 66), we also included negative controls and positive controls in our study. As negative controls, we included filtration blanks (n = 3) and extraction blanks (n = 6). We also included PCR no-template controls as discussed further below. We included two different positive controls in triplicate (n = 6 total) in the analysis. The two positive controls were (1) genomic DNA extracted from swordfish tissue (*Xiphias gladius*) and (2) a mock community with equal mass concentration of DNA from 9 species of bony fishes (S1 Table). The mock community and the methods used to create it are described in more detail elsewhere [33].

We used a two-step PCR method [38] to amplify extracted eDNA as well as add a unique tag to each sample. For the first PCR amplification, we used a published fish-specific primer set targeting a hypervariable region of the mitochondrial DNA 12S rRNA gene [23]. The primer sequences were F-5’ GTCGGTAAAACTCGTGCCAGC and R-5’ CATAGTGGGGTATCTAATCCCAGTTTG, amplifying a *ca* 170 bp region. PCR reactions were carried out using 3 μL of DNA extract diluted 1:10 (see “Inhibition testing” below), 0.4 μL of 10 μM forward and reverse primer (C_f_ = 0.2 μM), 10 μL of HotStarTaq Plus Master Mix (Qiagen, USA), and 6.2 μL of molecular-biology-grade water (Sigma-Aldrich, USA) for a total PCR reaction volume of 20 μL. We used eight-strip PCR tubes with individual caps to prevent cross contamination between samples. Each DNA extract (environmental samples, positive controls, and filtration and extraction blanks) (n = 81) was amplified in triplicate. A no template control (NTC) using molecular-biology-grade water in lieu of DNA template was included for each DNA extract to monitor for contamination in the master mix. A total of 81 NTCs were run; 81 NTCs were needed as 81 mastermixes (each with a unique set of tagged primers) were used in the second PCR (see below) and a NTC was needed for each mastermix. Thermal conditions for the first PCR amplification were 95°C for 5 min followed by 40 cycles of 95°C for 15 s, 55°C for 30 s and 72°C for 30 s.

After amplification, triplicate PCR products were pooled and visualized on a 1.5% agarose gel stained with ethidium bromide to confirm the presence of the target band and confirm no amplification in the NTCs. Pooled PCR products were cleaned using the Agencourt AMPure XP bead system (Beckman Coulter, USA), which removes primer dimers by size selection; cleaned products were quantified using the QUBIT DSDNA HS ASSAY (data not shown). The PCR product from the first amplification was then used as the template for the second PCR amplification. Despite the lack of amplification in NTCs, we included the NTCs in downstream processing as template for the second PCR amplification. This is a conservative approach to ensure that no contamination was present, even if no product was visualized in a gel. Similarly, none of the filtration or extraction blanks showed amplification in the gel visualization, but we carried these through the method with the other PCR products.

The second PCR amplification used the same primers listed above, but with the addition of 6 bp indices on the 5’ ends of the primers to allow concurrent sequencing of multiple samples. The tag sequences were used in a previous study [33] and were designed with a Hamming distance of at least three bases between tags and were preceded by NNN (S2 Table) [39]. The same tag was added to both the forward and reverse primer used to amplify each sample in order to reduce tag jumping [40]. The second PCR reactions were carried out in triplicate using 3 μL of the PCR product from the first PCR as template, 0.4 μL of 10 μM tagged forward and reverse primers unique for each sample (C_f_ = 0.2 μM), 10 μL of HotStarTaq Plus Master Mix (Qiagen, USA), and 6.2 μL of molecular-biology-grade water (Sigma-Aldrich, USA) for a total reaction volume of 20 μl. Thermal conditions for the second PCR amplification were 95°C for 5 min followed by 20 cycles of 95°C for 15 s, 57°C for 30 s and 72°C for 30 s.

After the second amplification, triplicate PCR products were pooled, visualized, cleaned, and quantified using the methods described above for the first PCR amplification. None of the negative controls showed amplification (no band in the gel) but were still prepared for sequencing. Similarly, none of the NTCs showed amplification after the first or second PCR amplification. We pooled individual NTCs together so that the pooled NTC could be included on the sequencing run (as described below). We prepared environmental samples, positive controls, negative controls, and the pooled NTC (hereafter “NTC”) for sequencing (n = 82).

#### Inhibition testing

Before amplification, a subset of samples was selected to test for inhibition by performing a series of dilutions. Samples were amplified using the untagged primers (first step PCR amplification) at the following dilutions: 1:1, 1:10, 1:50, 1:100. Based on the results of the test (data not shown), all samples were diluted 1:10 before amplification in order to reduce PCR inhibition.

### Library preparation and DNA sequencing

The tagged products from the second PCR amplification (environmental samples, positive controls, negative controls, and the NTC) were combined into 3 pools to create 3 libraries. As these pools were designed to have similar volumes, 50 ng of DNA from each sample was added to each pool. For the filter blanks, extraction blanks, and NTC, we added the average volume added to the pool for the environmental samples/positive controls because their DNA concentrations were too low to quantify. The total concentrations of DNA for each of the 3 pools were quantified using the QUBIT DSDNA HS ASSAY (data not shown), and 250 ng of each pool was used for library preparation with the KAPA Hyper Prep kit (KAPA Biosystems, USA). Each library had a NEXTFLEX DNA barcode (BIOO Scientific, USA) added during the library preparation containing a unique 6 bp identifier as well as the Illumina adapter sequence, resulting in 3 barcoded libraries. The 3 libraries were then combined with an equal mass of DNA (227 ng per library). The final concentration of the 3 combined libraries was 22.2 ng/μl. We used a Bioanalyzer with High Sensitivity DNA assay (Agilent Technologies, USA) to confirm library size and concentration. We sequenced the 3 libraries on an Illumina MiSeq platform at the Stanford Functional Genomics Facility using 2x250 paired-end sequencing and adding a 20% Phi-X spike-in control.

#### Sequence analysis

Bioinformatic analyses were performed using a Unix shell script [33]. Paired-end reads were merged using PEAR (v0.9.6) [41] with the following parameters: maximum assembly length = 251, minimum assembly length = 150, quality score threshold = 15, and p-value = 0.01. The reads were filtered using the fastq_filter command in USEARCH (v1.8.0) [42] for a minimum sequence length of 251 and expected errors per read of 0.5. Sequences were demultiplexed and only retained if the tag added during amplification was found on both the forward and reverse read to eliminate samples with tag jumping [38,43]. Primers were removed using cutadapt (v1.8.3) and singleton reads were removed. Sequences were clustered into OTUs using SWARM (v2.1.5) [44] with a cluster radius of 1; OTUs less abundant than 0.005% were removed [45].

We then compared the number of reads for each OTU in the filtration blanks, extraction blanks, and NTC with the number of reads in the environmental samples and positive controls. For each OTU for which there were reads in the blanks and/or NTC, we subtracted the maximum read number among the blanks and NTC from the read number in the environmental samples and positive controls (S3 Table).

OTUs were then annotated by comparing a representative sequence of each OTU to sequences deposited in the National Center for Biotechnology Information (NCBI) nucleotide (nt) database (downloaded January 4^th^, 2017) using BLAST+ (2.2.31+) [46]. The following parameters were used: percent identity = 97%, word size = 30, e value = 1e-20. The percent identity cut-off of 97% is the parameter used by the authors who developed the primers as well as another published marine metabarcoding study [2,23]. As representative sequences from OTUs often hit multiple entries in the NCBI database, we used the “taxize” package in R [47] to summarize the BLAST+ results, and we used the entry with the lowest e-value for each OTU to assign taxonomy. In the case that an OTU matched multiple entries in the nt database with the same percent identity and e-value, we used the lowest common taxonomic rank to annotate the OTU. For example, if an OTU matched two species with the equal parameters, no distinction could be made between which species the OTU represented so we assigned a genus level annotation. For this paper, we define a “taxon” as an OTU that was annotated using the set parameters described above.

We then removed OTUs classified as non-vertebrates (e.g., Gammaproteobacteria) and non-marine vertebrates (e.g., *Canis lupis* or *Homo sapiens*) (S4 Table). To account for uneven sequencing depths, we rarefied each sample (environmental and positive controls) to 45,000 reads using the “rrarefy” function in the R package vegan [48]. We chose 45,000 as 63/66 environmental samples had >45,000 reads (S1 Fig). The 3 environmental samples with less than 45,000 reads had the majority of reads assigned to non-marine vertebrates (i.e., *Sus scrofa*, *Homo sapiens*). These three environmental samples (3F-0m-Rep3, 5F-0m-Rep1, and 5F-0m-Rep3) were removed from subsequent analyses.

#### Statistical analysis

We investigated whether vertebrate community composition, as inferred using eDNA metabarcoding, was related to sampling depth or water column depth using a 2-way crossed ANOSIM implemented in the software package Plymouth Routines in Multivariate Ecological Research (PRIMER6) [49]. ANOSIM used Jaccard distance matrices generated using presence/absence data at the OTU level. The null hypotheses were that the distances between samples collected at the same sampling depth are smaller or equal to the distances between samples collected at different depths; and that the distances between samples collected at stations with similar water column depth are smaller or equal to the distances between samples collected at stations with different water column depths. Samples were either collected at the “surface” (0 m) or at “subsurface” (20 or 40 m), and at stations either in a “canyon” (>200 m deep) or on the “shelf” (<200 m deep). We used Kruskal-Wallis tests and Spearman rank correlations with presence/absence at the OTU level to investigate relationships among biological replicates (only at sampling depths with three biological replicates after removing samples with <45,000 reads). A p-value of 0.05 served as a cut off for statistical significance.

#### Census of marine vertebrate taxa and cross-verification

We compared taxa identified using eDNA metabarcoding to a historical record of taxa in MBNMS to determine if eDNA metabarcoding gives a reasonable census of marine vertebrates in the surveyed region. Annotation of representative OTU sequences varied in taxonomic rank. We took assignments at the species, genus, tribe, subfamily, and family level and generated a list of all taxa using their family designation. We used the “Checklist of Fishes Known to Occur in Monterey Bay National Marine Sanctuary” [50] compiled in 2013 from a variety of guidebooks, local experts, and field studies, as well as a “Site Characterization” published by MBNMS managers for pinnipeds and cetaceans [51] as the historical record.

## Results

### Raw sequence processing

Across the 82 samples, including positive controls, blanks, and the NTC, the sequencing runs produced 14,928,120 reads with an average error rate of 1.65%; 92.53% of reads had a Q score of ≥30. After merging paired-end reads, fastq quality filtering, identifying tags and adapters, and removing singletons, 6,010,859 high quality reads remained in the environmental samples, positive controls, and negative controls (Table 2). The average number of high quality reads per environmental sample was 85,240 and ranged from 45,829 to 127,269. The blanks and NTC (n = 10) had between 24 and 11,421 reads (median = 47) (S3 Table). SWARM generated 4,775 OTUs across the environmental samples, positive controls, blanks, and NTC. We subtracted the maximum number of reads for each OTU found in the negative controls from the positive controls and environmental samples and we removed any OTUs annotated to non-vertebrates and non-marine vertebrates. The reads from positive controls and environmental samples were then rarefied to 45,000 per sample to account for unequal sequencing depths (S1 Fig). 3,530 OTUs remained across the environmental samples, which we annotated to the lowest taxonomic rank. Of the 3,530 OTUs, 1,165 were annotated as 92 unique marine vertebrate taxa using the NCBI database (S4 Table). Although 2,365 OTUs were not annotated, they represent just 4.4% of the rarefied reads.

**Table 2:** Number of sequencing reads retained during data processing.

Data Processing Step	Number of Reads	Number of OTUs
Total sequencing reads from MiSeq run	14,928,120	
Merging of paired-end reads	9,157,301	
Fastq quality filtering	9,144,404	
Removal of reads with missing or mismatching tags	6,552,189	
Removal of reads without primers	6,291,698	
Singleton removal, cluster OTUs using SWARM	6,010,859	4,775
Subtract maximum number of reads for each OTU found in negative controls	5,953,367	4,769
Rarefy to 45,000 reads per sample	3,240,000	4,775
Remove positive control samples	2,970,000	3,617
Remove any non-marine or non-vertebrate OTUs	2,866,182	3,530

### Positive and negative controls

Three types of negative controls samples were sequenced; these included 3 filter blanks, 6 extraction blanks, and a representative NTC for a total of 10 negative controls. The negative controls had orders of magnitude lower number of reads than the environmental samples and positive controls (S3 Table, median = 47 for negative controls compared to 84,595 for environmental samples). To be conservative, for each OTU, if one or more negative control contained reads for that OTU, we subtracted the maximum number of reads found in the negative control from the number of reads recorded for that OTU in each environmental sample and positive control, while not letting the number of reads fall below 0. This affected a total of 61 unique OTUs (S3 Table). We made this adjustment before rarifying the data and before any statistical analyses. We also completed all the analyses described in this paper with unadjusted data (ignoring the results from the negative controls) and the results of the analyses did not change (data not shown).

We sequenced two types of positive controls each in triplicate: DNA extracted from swordfish (*Xiphias gladius)* tissue and a mock “community” constructed from DNA extracted from the tissue of 9 fish (S1 Table). The three replicates of the swordfish control produced 134,999 reads out of 135,000 (3 x 45,000) reads assigned to *Xiphias gladius*; 1 read was assigned to *Chilara taylori*, suggesting extremely low cross contamination or sequencing errors occurred in these samples. Combining reads from the three mock community replicates, eDNA metabarcoding identified 8 of the 9 mock community taxa. No reads were assigned to the 9^th^ taxon: *Paralichthys* (large-tooth flounder). Of all the sequencing reads from the mock community samples, 25.5% were not annotated using the criteria described in the methods section. There were no reads assigned to taxa not present in the mock community. Although DNA from each of the 9 taxa were combined in equal mass concentrations to construct the standard, the relative abundance of the taxa inferred from the number of sequencing reads do not reflect equal proportions (S1 Table) [33].

### Difference among biological replicates

**Fig 2:**
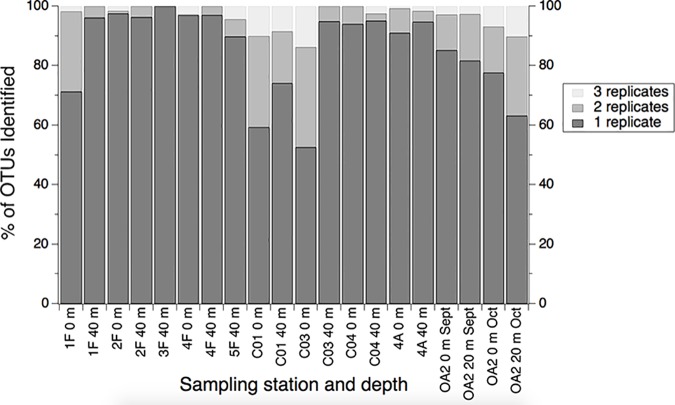
Percent of OTUs identified in 1, 2 or 3 of the biological replicates collected at each station/sampling depth. Samples are labeled with station (i.e., 1F, 2F, etc.) followed by the sampling depth (i.e., 0 m, 20 m, 40 m). 3F-0 m and 5F-0 m are not shown because they do not have complete sets of three replicates after rarefying.

We found that biological replicates did not identify the same OTUs (Fig 2). The majority of OTUs (over 52%) identified at each sampling depth was found in only one of three biological replicates; between 0 and 13.7% of OTUs were found in all three replicates. There was a significant negative correlation between the total number of OTUs identified at a sampling depth and the percent of the OTUs found in just one biological replicate (Spearman rho = -0.54, p = 0.014, n = 20). However, there is no difference in the percent of total OTUs found in only one biological replicate between samples collected at surface versus those collected at subsurface (Kruskal-Wallis test, p = 0.21, H = 1.57); the median percent for surface samples was 85.23% and for subsurface samples was 94.96%. There is also no difference in the percent of OTUs found in only a single biological replicate and the depth of the water column where the sample was collected (Kruskal-Wallis test, p = 0.16, H = 1.98); the median percent of OTUs was 85.23% in the shelf samples versus 95.12% in the canyon samples.

We recognize that rare OTUs might not be found in all three biological replicates. However, even if we consider just the 100 most abundant OTUs across all samples, there is still a large percentage of OTUs found in just one biological replicate (S2 Fig, range: 0% to 100%, median: 61.90%).

### Taxa/Families identified via eDNA metabarcoding

We identified 92 marine vertebrate taxa annotated at the taxonomic rank of family or lower in the environmental samples. These taxa were annotated at variable ranks: 72 were identified to species level, 10 to genus level, 3 to subfamily level, and 7 to family level. The 92 taxa represent 33 unique marine vertebrate families (Table 3). Between 3–21 unique families were detected at each station (across both sampling depths and all biological replicates) (S3 Fig, S5 Table), and the four most common were Myctophidae, Paralichthyidae, Scombridae, and Sebastidae (Table 4), which are all families with species well known to be in the region.

**Table 3:** Taxa identified using edna metabarcoding.

Class	Family (Common Name)	Family (f), Subfamily (sf), Tribe (t), *Genus (g)*, *Species (s)* Annotated
Chondrichthyes	Lamnidae (Mackerel Sharks)	Carcharodon carcharias* *(s)*
	Squalidae (Dogfish Sharks)	Squalus suckleyi* *(s)*
Actinopterygii	Engraulidae (Anchovy)	Engraulis mordax* *(s)*
	Clupeidae (Herrings, Shads, Sardines)	Clupea** *(g)*, Sardiops** *(g)*, Clupea harengus** *(s)*, Clupea pallasii* *(s)*, Sardinops melanostictus** *(s)*
	Salmonidae (Salmon, Trout, Chars)	Oncorhynchus kisutch* *(s)*
	Microstomatidae (Pencilsmelts)	Nansenia sanrikuensis** *(s)*
	Synodontidae (Lizardfish)	Synodus lucioceps* *(s)*
	Myctophidae (Lanternfish)	Myctophidae*** *(f)*, Diaphus theta* *(s)*, Lampanyctus tenuiformis* *(s)*, Nannobrachium fernae** *(s)*, Stenobrachius leucopsarus* *(s)*, Symbolophorus californiensis* *(s)*, Triphoturus mexicanus* *(s)*
	Ophidiidae (Cusk-eels)	Chilara taylori* *(s)*
	Bythitidae (Brotulas)	Brosmophycis marginata* *(s)*
	Embiotocidae (Surfperches)	Embiotocidae*** *(f)*, Brachyistius frenatus* *(s)*, Cymatogaster aggregata* *(s)*, Embiotoca jacksoni* *(s)*, Embiotoca lateralis* *(s)*, Hyperprosopon anale* *(s)*, Rhacochilus vacca** *(s)*
	Gobiesocidae (Clingfish)	Gobiesox maeandricus* *(s)*
	Scomberesocidae (Sauries)	Cololabis saira* *(s)*
	Carangidae (Jacks, Pompanos, Mackerels)	Trachurus** *(g)*, Decapterus macrosoma** *(s)*, Seriola lalandi* *(s)*, Trachurus symmetricus* *(s)*
	Paralichthyidae (Sand Flounders)	Citharichthys sordidus* *(s)*, Citharichthys stigmaeus* *(s)*, Citharichthys xanthostigma* *(s)*, Etropus microstomus*** *(s)*
	Pleuronectidae (Righteye Flounders)	Pleuronectidae*** (f), Hippoglossus** *(g)*, Eopsetta jordani* *(s)*, Pleuronichthys decurrens* *(s)*
	Syngnathidae (Seahorses, Pipefish)	Syngnathus leptorhynchus* *(s)*
	Scombridae (Mackerels, Tunas, Bonitos)	Scombrinae*** (sf), Scomber** *(g)*, Thunnus** *(g)*, Euthynnus alletteratus** *(s)*, Scomber australasicus** *(s)*, Scomber colias** *(s)*
	Tetragonuridae (Squaretails)	Tetragonurus cuvieri* *(s)*
	Sebastidae (Rockfish, Rock Perches)	Sebastidae*** (f), Sebastinae*** (sf), Sebastes** *(g)*, Sebastes auriculatus* *(s)*, Sebastes babcocki* *(s)*, Sebastes diploproa* *(s)*, Sebastes entomelas* *(s)*, Sebastes koreanus** *(s)*, Sebastes mystinus* *(s)*, Sebastes oblongus** *(s)*, Sebastes paucispinis* *(s)*, Sebastolobus macrochir** *(s)*
	Stichaeidae (Pricklebacks)	Opisthocentrinae*** (sf), Anisarchus medius*** *(s)*, Askoldia variegata*** *(s)*, Plectobranchus evides* *(s)*, Stichaeopsis epallax*** *(s)*
	Cryptacanthodidae (Wrymouths)	*Cryptacanthodes bergi (s)*, *Cryptacanthodes giganteus (s)*
	Pholidae (Gunnels)	Apodichthys flavidus* *(s)*
	Anarhichadidae (Wolffish)	Anarrhichthys ocellatus* *(s)*
	Zaniolepididae (Combfish)	Zaniolepis** *(g)*
	Hexagrammidae (Greenlings)	Hexagrammidae*** (f), Hexagrammos** *(g)*, Hexagrammos agrammus** *(s)*, Hexagrammos decagrammus* *(s)*, Hexagrammos otakii** *(s)*, Ophiodon elongatus* *(s)*, Oxylebius pictus* *(s)*
	Cottidae (Sculpins)	Hemilepidotus** *(g)*, Hemilepidotus spinosus* *(s)*, Leptocottus armatus* *(s)*, Scorpaenichthys marmoratus* *(s)*
	Sciaenidae (Drums)	Genyonemus lineatus* *(s)*
	Molidae (Ocean Sunfish)	Mola mola* *(s)*
Mammalia	Phocidae (Earless Seals)	*Mirounga angustirostris*^ *(s)*, *Phoca vitulina*^ *(s)*
	Otariidae (Eared Seals)	Otariidae^^^ (f), *Eumetopias jubatus*^ *(s)*, *Zalophus californianus*^ *(s)*
	Balaenopteridae (Rorquals)	*Megaptera novaeangliae*^ *(s)*
	Delphinidae (Oceanic Dolphins)	Delphinidae^^^ (f), *Grampus griseus*^ (s)

The 92 annotated taxa are named in the third column from the left. The class and family are shown in the first and second column, respectively.

* Indicates species on "Checklist of Fishes Known to Occur in Monterey Bay National Marine Sanctuary"

** Indicates genus on "Checklist of Fishes Known to Occur in Monterey Bay National Marine Sanctuary"

*** Indicates family on "Checklist of Fishes Known to Occur in Monterey Bay National Marine Sanctuary"

^ Indicates species on list of MBNMS Site Characterization Species List

^^^ Indicates family on list of MBNMS Site Characterization Species List

**Table 4:** Presence/Absence of 33 families at each station identified during the study across biological replicates and sampling depths.

Class	Family (Common Name)	Station										
		1F	2F	3F	4A	4F	5F	C01	C03	C04	OA2-SEPT	OA2-OCT
Chondrichthyes	Lamnidae (Mackerel Sharks)	0	0	0	0	0	0	0	0	0	1	0
	Squalidae (Dogfish Sharks)	0	0	0	0	0	1	0	0	0	0	0
Actinopterygii	*Engraulidae (Anchovy)*	0	1	0	1	0	1	1	1	1	0	1
	*Clupeidae (Herrings*, *Shads*, *Sardines)*	0	0	0	1	0	0	1	0	1	1	1
	Salmonidae (Salmon, Trout, Chars)	0	0	0	1	0	0	0	0	0	0	0
	Microstomatidae (Pencilsmelts)	0	0	1	0	0	0	0	1	0	0	0
	Synodontidae (Lizardfish)	0	0	0	0	0	0	1	0	0	0	0
	*Myctophidae (Lanternfish)*	1	1	1	1	0	0	1	1	1	1	0
	Ophidiidae (Cusk-eels)	0	0	0	0	1	0	0	0	0	0	1
	Bythitidae (Brotulas)	0	0	0	0	0	0	0	0	0	1	0
	Embiotocidae (Surfperches)	0	0	0	1	0	0	1	0	0	1	1
	Gobiesocidae (Clingfish)	0	0	0	0	0	0	0	0	0	0	1
	*Scomberesocidae (Sauries)*	1	1	0	0	1	0	0	0	0	0	0
	*Carangidae (Jacks*, *Pompanos*, *Mackerels)*	0	0	0	1	0	0	1	0	1	1	1
	*Paralichthyidae (Sand Flounders)*	1	1	0	1	1	1	1	1	1	1	1
	Pleuronectidae (Righteye Flounders)	0	0	0	0	0	0	1	0	0	1	1
	Syngnathidae (Seahorses, Pipefish)	0	0	0	0	0	0	0	0	0	0	1
	*Scombridae (Mackerels*, *Tunas*, *Bonitos)*	1	1	1	1	0	1	1	1	0	1	1
	Tetragonuridae (Squaretails)	0	0	1	0	0	0	0	0	0	0	0
	*Sebastidae (Rockfishes*, *Rock Perches)*	1	0	1	1	1	0	1	1	0	1	1
	Stichaeidae (Pricklebacks)	0	0	0	0	0	0	0	0	0	1	1
	Cryptacanthodidae (Wrymouths)	0	0	0	0	0	0	0	0	0	1	1
	Pholidae (Gunnels)	0	0	0	0	0	0	0	0	0	0	1
	Anarhichadidae (Wolffish)	0	0	0	0	0	0	0	0	0	1	1
	Zaniolepididae (Combfish)	0	0	0	0	0	0	0	0	0	1	0
	*Hexagrammidae (Greenlings)*	0	1	1	0	1	0	1	0	0	1	1
	Cottidae (Sculpins)	0	0	0	0	0	0	1	0	0	1	1
	Sciaenidae (Drums)	0	0	0	0	0	0	1	0	0	1	1
	Molidae (Ocean Sunfish)	0	0	0	1	0	0	0	0	0	0	1
Mammalia	Phocidae (Earless Seals)	0	0	0	0	0	0	0	0	0	1	1
	*Otariidae (Eared Seals)*	0	0	0	0	0	0	1	0	1	1	1
	Balaenopteridae (Rorquals)	0	0	0	0	0	0	1	0	0	1	0
	*Delphinidae (Oceanic Dolphins)*	0	0	1	0	0	0	1	0	1	0	0
**Total # of Families per Location**		**5**	**6**	**7**	**10**	**5**	**4**	**16**	**6**	**7**	**20**	**21**

1 = present, 0 = absent. Light shading indicates families found only at stations on the shelf (<200 m water column depth). Dark shading indicates families found only at stations in a canyon (>200 m water column depth). *Italics* indicate families found at both locations.

**Fig 3:**
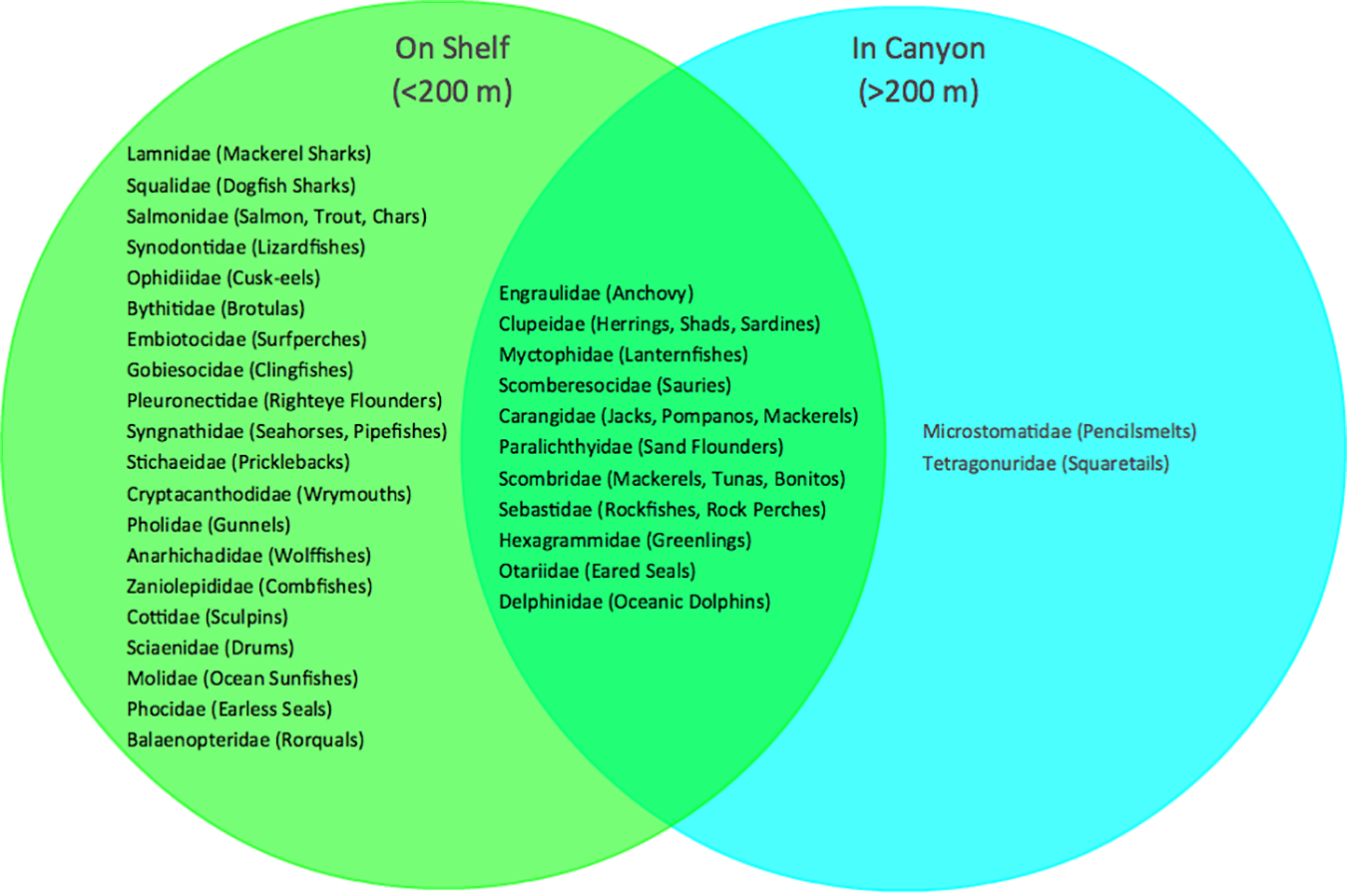
Families identified using eDNA metabarcoding in samples collected at stations on the continental shelf (water column depth < 200 m) and stations in a canyon (water column depth >200 m). 20 families were only found on the shelf, 2 were only found in a canyon, and 11 were found at both.

**Fig 4:**
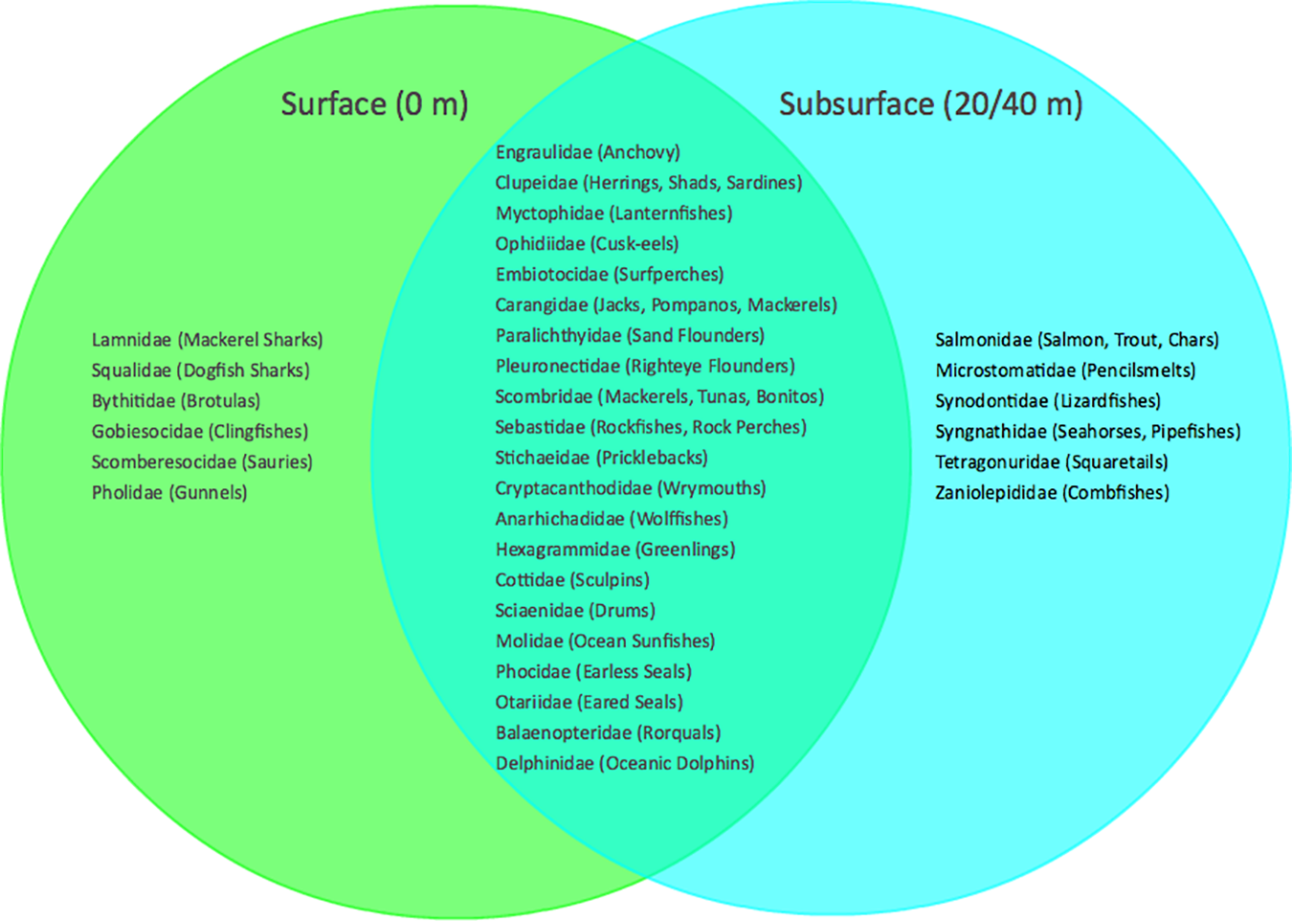
Families identified using eDNA metabarcoding in samples collected at surface and at subsurface (20/40 m). 6 families were only found when sampling at the surface of the water, 6 families were only found when sampling the subsurface (20/40 m), and 21 of 33 families were found both at surface and subsurface.

Station OA2, located on the continental shelf and inside the Año Nuevo State Marine Reserve, had the greatest number of families present (26 families across the 2 sampling days). This was also the shallowest of all sampling stations. Of the 33 families found across all stations and sampling depths, 20 were identified only at stations on the continental shelf, 2 were identified only at stations within a canyon, and 11 were found both at stations on the shelf and stations in a canyon (Fig 3). Similarly, 6 of the 35 families were only found in surface samples, 6 were only found in subsurface samples (20/40 m deep), and 21 were found in both surface and subsurface samples (Fig 4).

### Comparison of taxa identified using eDNA metabarcoding and those known to occur in MBNMS

Of the 33 marine vertebrate families identified using eDNA metabarcoding, 32 are known to occur within MBNMS (Table 3 and S6 Table). Cryptacanthodidae (wrymouths) was the only family not noted in the historical list that was identified in the sequences.

Of the 72 species level taxonomic assignments, 52 are known to occur within MBMNS. Most of the remaining species (18 of 20), although not on the MBMNS checklist, belong to genera or families represented on the checklist (Table 3 and S6 Table).

### Vertebrate community composition at different sampling and bottom depths of Monterey Bay

Samples collected at the same depth in the water column (n = 30) were more similar than samples collected at different depths (n = 33) (ANOSIM R = 0.059, p = 0.041). Also, samples collected at stations on the shelf (n = 34) or in a canyon (n = 29) were more similar to each other than to samples collected at stations with varying water column depths (R = 0.100, p = 0.002). The R values are small, despite the null hypotheses being rejected, suggesting that there is overlap in community composition among the samples.

## Discussion

### eDNA metabarcoding provides a realistic census of marine vertebrates

Thirty-two out of 33 families detected using eDNA metabarcoding are known to be present in MBNMS. The families have been documented in regional guidebooks and literature, identified in recent field surveys, or have been catalogued in ichthyology collections [50]. Cryptacanthodidae (wrymouths) is the only family detected by eDNA metabarcoding not known to occur in the area. The native habitats for the two species within Cryptacanthodidae (*Cryotacanthodes bergi* and *Cryptacanthodes giganteus*) occur in the Northwest Pacific for *C*. *bergi* and the Northeast Pacific extending down to Northern California for *C*. *giganteus*. It remains possible that Cryptacanthodidae have migrated into MBNMS since 2013 when the historical list of MBNMS organisms was last updated. It is also possible that the eDNA identifications of Cryptacanthodidae were false positives. If they were, the family-level false positive rate of eDNA metabarcoding in this study would be 3%. Other eDNA metabarcoding studies have reported false positive rates of 0 [34] to 8.3% [2].

The historical list of species present in MBNMS contains 146 families whose presence presumably varies over time and space. It is unlikely that all of these families were present during our sampling cruise, thus, we are not able to generate a false negative rate for this study. Studies using species-specific qPCR report false negative rates ranging from 0–8.2% [21,28,52], but most eDNA metabarcoding studies in environmental waters do not report false negative rates. Port et al. [33] reported an eDNA metabarcoding false negative rate of 8.3% using organism counts from a visual dive survey as the “true” census of marine vertebrates. However, the use of visual counts as the “gold standard” for biomonitoring has drawbacks as described by Kelly et al. [53].

There are differences in vertebrate community composition identified by eDNA metabarcoding between the surface and subsurface waters, and between neritic (<200 m depth) stations versus deepwater stations in Monterey canyons. Certain taxa were found in deep waters within canyons and others remain close to shore in shallow waters. For example, we identified taxa in the family Cottidae only at stations on the shelf; the family is known to be found in shallow waters near shore [54]. Other families that were found only on the shelf are known to inhabit neritic, shallower waters, living in rocky areas or kelp forests [51,54]. We found the shallowest station (OA2) to have the highest biodiversity.

In addition to biodiversity changing across the topographical environments, we found differences in vertebrate communities in surface versus subsurface waters. During the cruise, the water column was stratified with a continuous, nearly linear trend between temperature and depth (|**Δ**T| between surface and subsurface sampling depths was, on average 2.7°C, and ranged from 1.4°C–3.6°C). The thermocline limits vertical mixing of eDNA in the water column and the environments above and below the thermocline represent different ecological niches. Thus the presence of the thermocline may explain the observation of different vertebrate taxa identified using eDNA metabarcoding at different sampling depths in the water column. However, the discrepancy between eDNA found at the surface and subsurface could potentially be due to physical properties of eDNA (i.e., its density or whether it is particle-association) or the latency in degradation between warmer waters and cooler waters at depth.

### Biological replicates identify different taxa

Previous eDNA studies have not included true biological replicates. Rather, they included pseudoreplicates (multiple subsamples from one large volume sample) [2,23,33], or pooled sequencing results from biological replicates [23]. The results from our study indicate that a single, unreplicated sample is not necessarily representative of the water being sampled. This may be due to eDNA not being homogenously mixed throughout the sampled water mass. We found a negative correlation between number of OTUs and the percent found in one replicate. This indicates that as more OTUs are identified in a sample, fewer OTUs are found in just one replicate. Until there is a better understanding of the spatial heterogeneity of eDNA in the water column, biological replicates should be collected for eDNA metabarcoding studies and combined when analyzing presence and/or absence data. Another possibility is that the difference between biological replicates could be influenced by the stochastic nature of the PCR process [15].

### eDNA metabarcoding limitations

False positives and false negatives are concerns for any biomonitoring method. Potential sources of false positives in eDNA metabarcoding include cross-contamination between samples or from positive controls, sequences being assigned to the wrong taxa, or a misidentification of a species deposited in the NCBI database [31,32]. False negatives might result from eDNA from an organism not being captured in the water sample, target eDNA not being amplified by universal primers, taxa with low numbers of sequencing reads being lost during rarefaction, or PCR amplification bias [6,31,32,55,56]. False negatives could also be a result of an OTU that is not annotated. Species occupancy models and statistical methods have been developed to account for false positives and false negatives when interpreting traditional biomonitoring data (i.e., data collected using fish trawls, electroshocking, and visual surveys) [57,58] and may also be useful for interpreting eDNA metabarcoding data [31,34,59–61]. However, these models require that species be identified using multiple detection methods or that false positive and negative detection rates are known a priori [59].

Of the 3,530 OTUs identified in the environmental samples, 2,150 did not have representative sequences matching entries in the NCBI nucleotide database. While those 2,150 OTUs only contain 4.3% of sequencing reads, the lack of matches in the database can be explained in part by a data gap in the repository with respect to fish, shark, and marine mammal mitochondrial genomes. As an example, teleost fish (fish of the infraclass Teleostei) are the largest known group of vertebrates with more than 27,000 species, making up 96% of bony fish of the superclass Osteichthyes [62]. As of August 2016, the database had 28,462 entries of the mitochondrial 12S rRNA gene of teleosts, but only ~8,000 unique species meaning that only about 30% of teleost species have sequences deposited in the database. Results from the mock community positive control illustrate that this can occur, as the taxon present in the mock community that was not identified using eDNA metabarcoding (*Paralichthys*) has entries in the database. We found that the most abundant OTU that was not annotated by our methods in the mock community matched an entry in the NCBI nt database for *Paralichthys olivaceus*, submitted by the Kyoto Aquarium at 95% identity (a lower percent identity cutoff than used in the present study).

Finally, results from the mock community positive control highlight the challenges of using the described eDNA metabarcoding in a quantitative manner. The relative proportions of sequencing reads in the mock community are different than original proportions (based on DNA mass) used to construct the community potentially owing to the high cycles of PCR used in this study. Previous eDNA metabarcoding studies of fish (one in freshwater mesocosms and one in marine environmental waters) have reported that taxa relative abundance is positively associated with taxa counts obtained using visual surveys [33,63]. More work is needed to explore how eDNA metabarcoding may be used to obtain quantitative information on taxa abundance [61].

## Conclusions

This observational study in MBNMS finds that marine vertebrate communities identified using eDNA metabarcoding varied with depth in the water column. In addition, the vertebrate community varied as expected with total water column depth as the transect extended from coastal waters into canyon realms. These findings expand our current knowledge of the spatial heterogeneity of vertebrate eDNA. It also highlights the importance of collecting biological replicates for eDNA metabarcoding. eDNA metabarcoding provides a realistic census of marine vertebrates in MBNMS, including 33 total families found across all replicates, sampling depths, and stations, of which 32 were known to be in MBNMS.

eDNA metabarcoding offers enormous potential for biomonitoring. Sampling collection is fairly straightforward, and filters can be archived at -80°C for extended periods of time. Preliminary studies have shown that eDNA metabarcoding can identify vertebrate species missed by traditional monitoring methods and sample vertical distributions that would otherwise not be possible with traditional techniques. Moreover, eDNA metabarcoding can be used at finer temporal and spatial resolution compared to traditional biomonitoring methods to document changes in biodiversity over seasonal and annual cycles, and over topographic gradients.

## Supporting information

S1 Fig Rarefaction curves for environmental samples. Rarefaction curves for all environmental samples. Vertical line highlights 45,000 reads. Horizontal lines show number of OTUs per sample with 45,000 reads.
(TIFF)

S2 Fig Percent of top 100 OTUs (in terms of number of sequences across all samples) identified in 1, 2 or 3 of the biological replicates collected at each station/sampling depth. Samples are labeled with station (i.e., 1F, 2F, etc.) followed by the sampling depth (i.e., 0 M, 20 M, 40 M).
(TIFF)

S3 Fig Number of families found at each station across all biological replicates and sampling depths. Isobaths are labeled with their depth in meters.
(TIFF)

S1 Table Composition of mock community used as positive control and sequencing reads annotated to taxa in mock community and tissue sample positive controls. (XLSX)

S2 Table Sequences of tagged primers used for second PCR amplification. (XLSX)

S3 Table Sequence counts in negative control samples and representative NTC sample with annotations. "EB" stands for extraction blank. "FB" stands for filter blank.
(XLSX)

S4 Table Complete list of taxa before and after removing reads assigned to non-vertebrates and non-marine vertebrates. (XLSX)

S5 Table Number of total OTUs, annotated OTUs, and families found at each sampling station. (XLSX)

S6 Table Complete hierarchy for taxa found using eDNA metabarcoding compared to what is known to occur in the MBNMS. (XLSX)
