# Supplementary figures and images for: Biomonitoring of marine vertebrates in Monterey Bay using eDNA metabarcoding
Source: PLoS One. 2017 Apr 25;12(4):e0176343. doi: 10.1371/journal.pone.0176343 (PMC5404852; doi:10.1371/journal.pone.0176343)

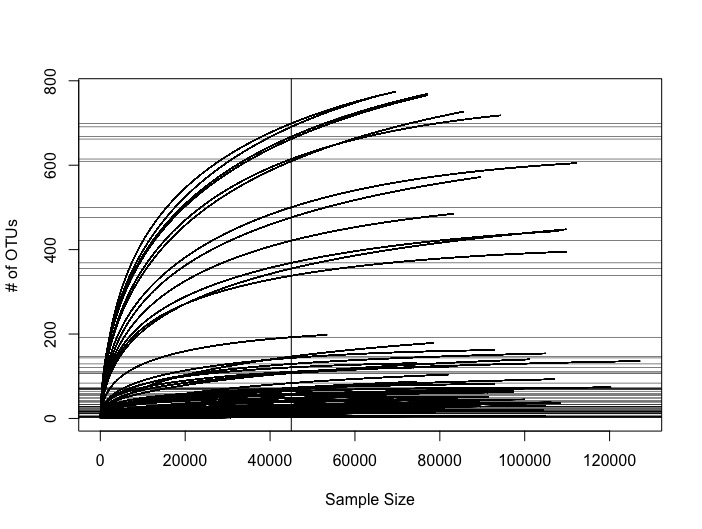

Supplement: S1 Fig — Rarefaction curves for all environmental samples. Vertical line highlights 45,000 reads. Horizontal lines show number of OTUs per sample with 45,000 reads. (TIFF) [file pone.0176343.s001.tiff]

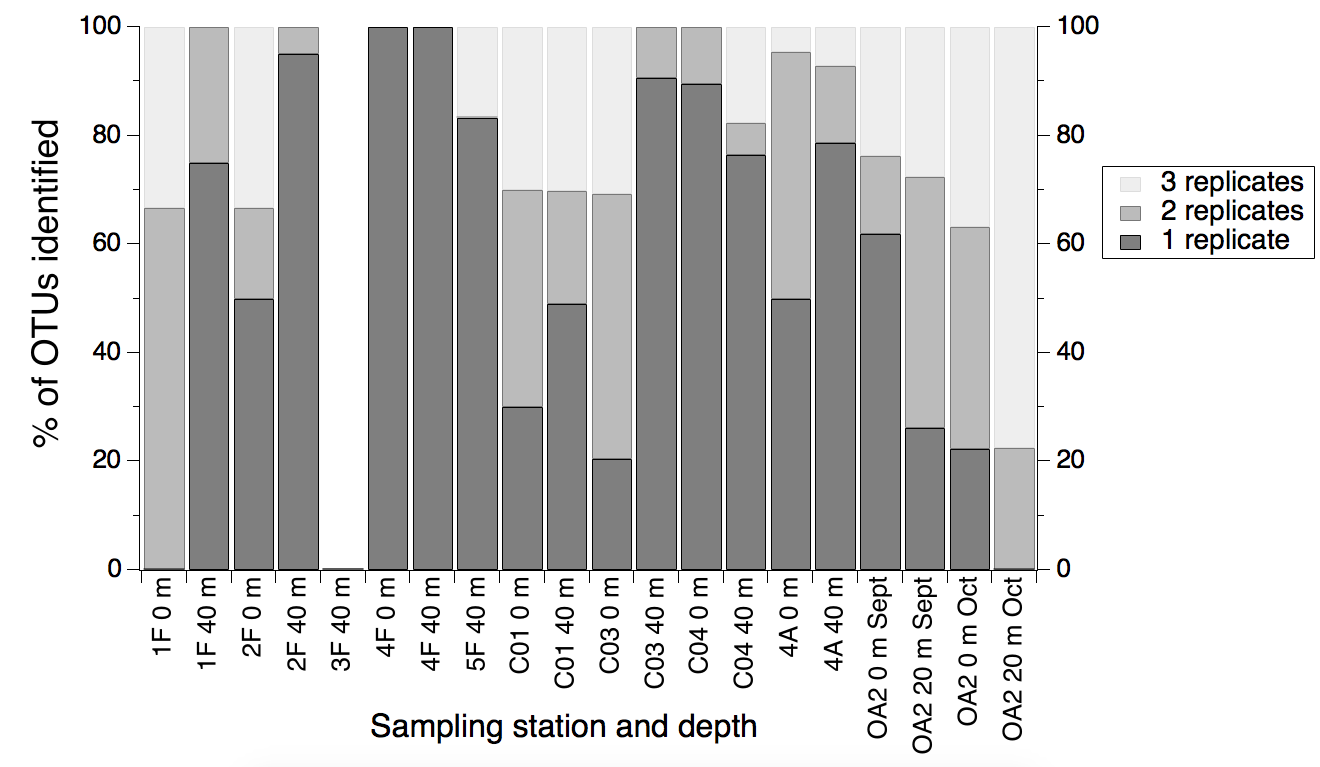

Supplement: S2 Fig — Samples are labeled with station (i.e., 1F, 2F, etc.) followed by the sampling depth (i.e., 0 M, 20 M, 40 M). (TIFF) [file pone.0176343.s002.tiff]

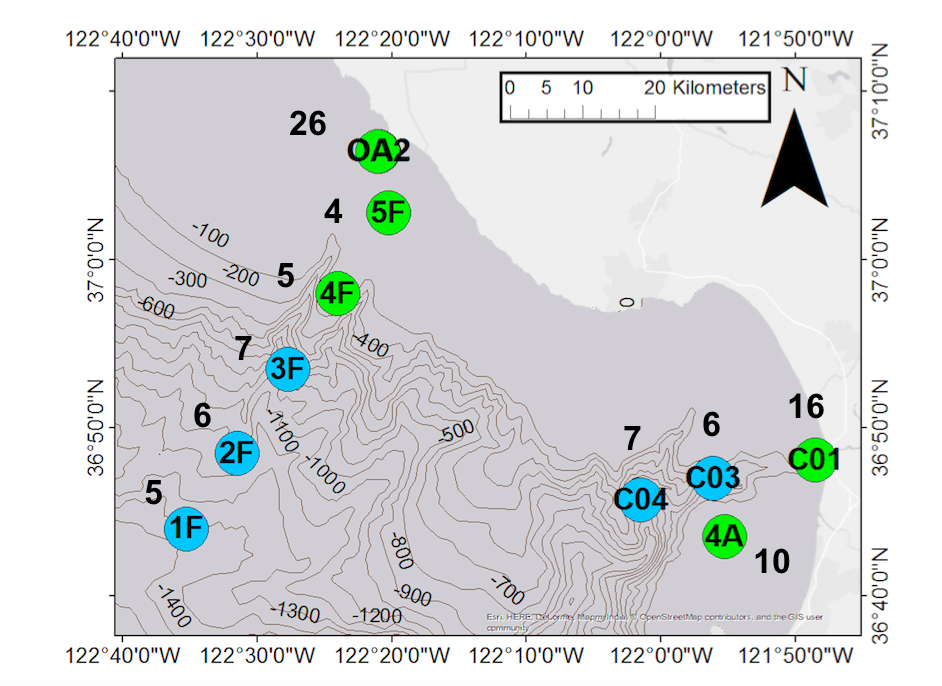

Supplement: S3 Fig — Isobaths are labeled with their depth in meters. (TIFF) [file pone.0176343.s003.tiff]
